# The Convergence of Antimicrobial Resistance and Virulence in *Streptococcus pneumoniae*: A Molecular and Clinical Perspective

**DOI:** 10.3390/microorganisms14071451

**Published:** 2026-06-30

**Authors:** Jorge Almeida, Kenichi Takeshita, Alejandra Ramirez-Villalva, Ana G. Jop Vidal, Pedro Alejandro Fong-Coronado, Javian E. Ervin, Gloria M. Castañeda-Ruelas, Jorge E. Vidal

**Affiliations:** 1 Professional School of Pharmaceutical Biological Chemistry, University of Ixtlahuaca CUI, Ixtlahuaca 50740, Estado de México, Mexico; angel.almeida@uicui.edu.mx (J.A.); alejandra.ramirez@uicui.edu.mx (A.R.-V.); 2 Department of Cell and Molecular Biology, University of Mississippi Medical Center, Jackson, MS 39216, USA; ktakeshita@umc.edu (K.T.); avidal@umc.edu (A.G.J.V.); pfong@umc.edu (P.A.F.-C.); jervin1@umc.edu (J.E.E.); 3 Biomedical Sciences Master of Science Program, University of Mississippi Medical Center, Jackson, MS 39216, USA; 4 Faculty of Chemical and Biological Sciences, Autonomous University of Sinaloa, Culiacan 80013, Sinaloa, Mexico; gloria.ruelas@uas.edu.mx; 5 Center for Immunology and Microbial Research, University of Mississippi Medical Center, Jackson, MS 39216, USA

**Keywords:** *Streptococcus pneumoniae*, antimicrobial resistance (AMR), virulence factors

## Abstract

Antimicrobial resistance (AMR) and virulence have traditionally been viewed as competing traits in bacterial evolution due to fitness costs. However, *Streptococcus pneumoniae* has emerged as a paradigm of successful coevolution, with multidrug-resistant clones simultaneously maintaining or enhancing pathogenic potential. This review examines the molecular mechanisms, epidemiological patterns, and clinical consequences of the convergence between AMR and virulence in *Streptococcus pneumoniae*. Resistance to β-lactams is driven by mosaic penicillin-binding protein genes (*pbp1a*, *pbp2b*, *pbp2x*), while macrolide resistance is mediated primarily by the *erm(B)* gene (MLS phenotype) and *mef(A/E)–msr(D)* genes encoding an efflux system. These determinants are frequently co-localized on integrative and conjugative elements, ICEs, (e.g., Tn916 family) within successful clonal complexes such as CC271/320 and lineages including ST320 and GPSC10. Contrary to the classical fitness cost hypothesis, compensatory epistasis, capsular recombination, metabolic adaptations, and intra-serotype phenotypic variation enable certain clones to combine high-level resistance to β-lactams, macrolides, and tetracyclines with enhanced colonization, biofilm formation, immune evasion, and invasive capacity. Post-pneumococcal conjugate vaccine (PCV) surveillance reveals the persistence and expansion of these high-risk lineages, contributing to treatment-refractory invasive pneumococcal disease (IPD), increased morbidity, and mortality. Although PCVs have reduced vaccine-type resistant strains in some settings, serotype replacement and emerging metabolic genotypes continue to drive adaptation. This review highlights the need for integrated genomic surveillance, novel therapeutics (e.g., omadacycline, lefamulin, endolysins), monoclonal antibodies, and next-generation vaccines targeting both resistance and conserved virulence determinants. A multifaceted strategy combining antimicrobial stewardship, strengthened surveillance, and innovative interventions is essential to curb the evolving threat of resistant and virulent *S. pneumoniae*.

## 1. Introduction

The discovery of antimicrobials in the early 20th century marked a pivotal advancement in modern medicine, dramatically reducing mortality from bacterial infections and enabling widespread therapeutic success in both human and veterinary contexts. Their use has since expanded to include prophylactic and growth-promoting applications in livestock and agriculture, particularly in resource-limited settings. However, the selective pressure imposed by inappropriate prescribing, overuse, diagnostic limitations, agricultural practices, and suboptimal patient adherence has accelerated the emergence and spread of antimicrobial resistance (AMR), a phenomenon observed as early as the introduction of penicillin [1,2].

AMR allows pathogenic microorganisms to persist and proliferate despite exposure to agents designed to inhibit or eliminate them, thereby rendering standard treatments ineffective and complicating clinical management. Recognized as a major global public health threat in the 21st century, AMR contributed substantially to morbidity and mortality in 2019, with an estimated 13.7 million infection-related deaths worldwide. Of these, approximately 7.7 million were associated with bacterial pathogens, and 4.95 million were linked to bacterial AMR, including 1.27 million deaths directly attributable to resistance [1,3].

AMR arises from evolutionary pressures on bacterial populations and includes mechanisms such as enzymatic inactivation (e.g., β-lactamases), target site modification, metabolic bypass, biofilm formation, reduced permeability, and efflux pump overexpression. Phenotypic methods (disk diffusion and broth microdilution) assess growth inhibition, while genotypic approaches detect resistance genes or mutations [4,5,6].

Consequently, when these mechanisms lead to widespread resistance, multidrug-resistant (MDR) pathogens are defined as non-susceptible to at least one agent in three or more antimicrobial classes or subclasses. Subcategories include extensively drug-resistant (XDR) and pandrug-resistant (PDR) isolates [7,8,9].

Since its inception in 2017, the World Health Organization (WHO) Bacterial Priority Pathogens List (BPPL) has served as a critical tool for prioritizing research, development, and investment in novel antimicrobials, while informing surveillance and control strategies for antimicrobial resistance. The 2024 update refines this prioritization, encompassing 24 resistant bacterial phenotypes across 15 families, grouped into critical, high, and medium categories. Notable refinements include the addition of macrolide-resistant *Streptococcus pneumoniae* to the medium-priority group, replacing the prior focus on penicillin-non-susceptible strains. This change reflects rising macrolide resistance and its substantial public health burden, particularly among vulnerable populations in low- and middle-income countries. This shift underscores the need for targeted interventions against community-acquired pathogens with evolving resistance profiles [10].

*S. pneumoniae* is a Gram-positive commensal of the upper respiratory tract that can cause mild respiratory tract infections (otitis media and sinusitis), and severe invasive pneumococcal disease (IPD), including pneumonia, meningitis, and sepsis [11,12]. These infections primarily affect children under five years of age, immunocompromised individuals, and older adults. Pneumococcal disease causes more than 300,000 deaths annually among children under five worldwide [13,14,15], while antimicrobial resistance has been associated with approximately 600,000 deaths attributable to *S. pneumoniae* [16]. This substantial burden is partially preventable through vaccination with pneumococcal conjugate vaccines (PCVs), which target the capsular polysaccharide (CPS)—a key virulence factor that enables immune evasion. The structural diversity of CPS allows differentiation into more than 100 serotypes, with currently licensed PCVs covering up to 21 of the most common disease-causing serotypes [17,18]. However, PCVs do not protect against non-encapsulated *S. pneumoniae* (NESpn) strains, which can also exhibit antimicrobial resistance [19]. Given these challenges in both therapeutic and vaccine development, continued research into novel strategies remains essential, including capsule-degrading enzymes, therapeutic protein-targeted antibodies, and reprogramming the innate and adaptive microbicidal pathway [20,21,22].

This review examines the interplay between bacterial virulence factors and antibiotic resistance mechanisms, highlighting their coevolutionary dynamics and implications for pathogenesis and therapeutic strategies.

## 2. Molecular Basis of Antimicrobial Resistance in *S. pneumoniae*

Currently, *S. pneumoniae* is classified by the WHO as a medium-priority pathogen due to its persistence, high disease burden, and increasing rates of macrolide resistance [12,23]. The evolution of antimicrobial resistance in *S. pneumoniae* is driven by multiple factors, including inappropriate antimicrobial use, serotype replacement, and vaccine-induced selective pressure [24]. Antimicrobial resistance in *S. pneumoniae* contributes to treatment failure, increased morbidity, and the need for more effective therapeutic options [25,26].

β-lactams are considered first-line antibiotics for the treatment of *S. pneumoniae infections.* Macrolides and fluoroquinolones are also included in therapeutic regimens for these infections [27]. Historically, *S. pneumoniae* was regarded as susceptible to available antimicrobial; however, the first case of penicillin-resistant *S. pneumoniae* was reported in 1965. Subsequently, the prevalence of resistant strains increased progressively during the latter half of the twentieth century [28]. Nowadays, *S. pneumoniae* exhibits an increasing prevalence of antimicrobial-resistant strains, with annual increases in resistance to ≥1 drug class (+0.9%), ≥2 drug classes (+1.8%), and macrolides (+5.0%). In addition, the organism displays intrinsic reduced susceptibility to aminoglycosides and intrinsic resistance to polymyxins (polypeptide antimicrobials) [29].

This bacterium has evolved diverse molecular mechanisms to evade the activity of the antimicrobials used to treat pneumococcal infections. In *S. pneumoniae,* resistance arises from a complex interplay of processes, including genetic mutations, horizontal gene transfer, and structural adaptations [30].

### 2.1. β-Lactams: Alterations in Penicillin-Binding Proteins (PBPs) and Mosaic Genes

β-lactams: Alterations in penicillin-binding proteins (PBPs) and mosaic genes. The β-lactam family comprises broad-spectrum antimicrobials, including penicillin, cephalosporins, and carbapenems [30]. These agents exert their antibacterial activity by targeting high-molecular-weight proteins known as penicillin-binding proteins (PBPs), which are essential enzymes involved in peptidoglycan biosynthesis. Inhibition of these enzymes disrupts cell wall synthesis, ultimately leading to bacterial lysis [31]. The widespread use of β-lactams and vaccine-driven serotype replacement has contributed to changes in the prevalence of β-lactam resistance [24].

*Streptococcus pneumoniae* develops resistance to β-lactams through structural alterations in PBPs, which reduce their affinity for these antibiotics and thereby diminish their efficacy [32]. PBPs contain domains with transpeptidase and carboxypeptidase activities that are essential for peptidoglycan cross-linking in the cell wall, and harbor three conserved motifs within their catalytic sites [24,32].

*Streptococcus pneumoniae* produces six PBPs (1a, 1b, 2a, 2b, 2x, and 3). However, PBP2x, PBP2b, and PBP1a are the primary determinants associated with clinical resistance to β-lactam [33,34]. The mechanism of resistance involves the formation of mosaic genes, particularly in *PBP2x*, *PBP2b*, and *PBP1a*, arising from horizontal gene transfer with closely related species such as *S. mitis* and *S. oralis* [34]. These mosaic genes contain sequence blocks that can differ from those of susceptible strains by approximately 14% to 23% [33].

Mutations that induce structural alterations in PBPs are a major mechanism of β-lactam resistance. Modifications in the active sites of *PBP2x* and *PBP2b* are typically responsible for low-level penicillin resistance [31]. High-level resistance, in contrast, requires cumulative alterations in the three principal PBPs-*PBP1a*, *PBP2x*, and *PBP2b* [24]. Mosaic variants of PBP2x and PBP1a are often sufficient to confer resistance to third-generation cephalosporins [24]. Additional contributing changes include point mutations in *PBP2x* that markedly reduce acylation efficiency, and mutations in *PBP2b* that decrease penicillin-binding affinity [34]. Overall, the formation of mosaic genes enables *S. pneumoniae* to acquire genetic elements, facilitating rapid adaptive evolution and evasion of β-lactam inhibitory activity [26,34].

Additional molecular factors contribute to the ability of *S. pneumoniae* to withstand β-lactam-induced stress. The *murMN* operon is a critical auxiliary determinant of high-level antimicrobial resistance. The *murMN* operon is a key auxiliary determinant of high-level β-lactam resistance [24]. *MurM* and *MurN* enzymes catalyze the synthesis of branched muropeptide precursors that are essential for altered PBPs to continue cell-wall biosynthesis in the presence of the antimicrobial. In resistant strains, deletion of *murM* results in complete loss of resistance [32].

In addition, the two-component CiaRH system—comprising the histidine kinase CiaH and its cognate response regulator CiaR—represents a PBP-independent mechanism of β-lactam resistance. CiaRH inhibits autolysis under cell wall stress induced by β-lactam antimicrobial. Specific mutations in *ciaH* have been shown to increase cephalosporin resistance. Furthermore, the CiaRH system regulates several downstream effectors, including the serine protease HtrA, as part of a broader antimicrobial stress-response network [24].

### 2.2. Macrolides: Target Modification (MLSB Phenotype) and Efflux (MEGA)

Macrolides are natural or semisynthetic antimicrobials widely used for the treatment of pneumococcal infections [35,36]. They are characterized by a macrolactone ring to which one or more deoxy-sugar moieties are attached. Based on the number of carbon atoms in the lactone ring, macrolides are classified as 14-membered (e.g., erythromycin, clarithromycin), 15-membered (e.g., azithromycin), or 16-membered (e.g., spiramycin) [27]. Structural variations within the ring significantly influence their pharmacokinetic properties and antibacterial activity [37].

Macrolides exert a primarily bacteriostatic effect against *S. pneumoniae* by inhibiting protein synthesis. They bind reversibly to domain V of the 23S rRNA in the 50S ribosomal subunit [34,38,39], disrupting peptide chain elongation through premature dissociation of peptidyl-tRNA from the ribosome. This leads to premature termination of translation and inhibition of bacterial growth [27,35]. The rising prevalence of macrolide-resistant *S. pneumoniae* poses a major clinical challenge [37,38].

Resistance to macrolides in *S. pneumoniae* is mainly mediated by two mechanisms: ribosomal target modification and active efflux. Target modification is the predominant mechanism and is encoded by the *erm(B)* gene, which produces a ribosomal methyltransferase. This enzyme methylates adenine residue A2058 in domain V of the 23S rRNA, blocking macrolide binding to the ribosome [36,37,40,41,42]. The resulting MLS phenotype confers cross-resistance to macrolides, lincosamide, and streptogramin B (MLS) [43,44]. Expression of this phenotype can be constitutive or inducible, with induction triggered by specific macrolides [45].

The second major mechanism involves active efflux mediated by the macrolide efflux genetic assembly (MEGA), a mobile genetic element (~5.4–5.5 kb) carrying the *mef(A/E)–mel* (also known as *msrD*) operon [36,37,40,41,42]. Transcription of this operon is driven by a macrolide-inducible promoter, resulting in the production of an efflux pump (Mef) and an ATP-binding cassette (ABC-F) ribosomal protection protein (Mel). The synergistic action of Mef and Mel actively exports 14- and 15-membered macrolides (e.g., erythromycin, clarithromycin, and azithromycin) out of the cell, producing the M phenotype [27,45,46].

Less common resistance mechanisms (~1.5%) involve point mutations in the 23S rRNA or in genes encoding ribosomal proteins L4 (*rplD*) and L22 (*rplV*), which alter the macrolide binding site [24].

### 2.3. Fluoroquinolones: Mutations of DNA Gyrase and Topoisomerase IV

Fluoroquinolones are synthetic antimicrobials derived from the quinolone scaffold, which consists of a bicyclic core with a carboxylic acid group at position 3 and a keto group at position 4. The addition of a fluorine atom at position 6 markedly enhances antibacterial potency and broadens the spectrum of activity [47]. Further substitutions at various positions modulate their pharmacological properties and antimicrobial spectrum [47,48]. Key representatives used in pneumococcal infections include ciprofloxacin, levofloxacin, moxifloxacin, gatifloxacin, gemifloxacin, and sitafloxacin [49].

Fluoroquinolones exert their bactericidal effect by inhibiting bacterial DNA replication. They bind to and stabilize ternary complexes formed between DNA and the two essential type II topoisomerases: DNA gyrase (GyrA/GyrB) and topoisomerase IV (ParC/ParE) [48,50]. In *S. pneumoniae*, topoisomerase IV is typically the primary target, while DNA gyrase serves as the secondary target [51]. This stabilization prevents replication fork progression and transcription, ultimately leading to double-strand DNA breaks and bacterial cell death [47,49].

The increasing use of fluoroquinolones has driven the emergence of resistant *S. pneumoniae* strains. Resistance is primarily mediated by stepwise mutations in the quinolone resistance-determining regions (QRDRs) of DNA gyrase and topoisomerase IV. Secondary mechanisms include reduced drug permeability, overexpression of multidrug efflux pumps, and, to a lesser extent, plasmid-mediated resistance [47].

Target-site mutations represent the dominant resistance mechanism [47]. They usually occur in a stepwise fashion: initial mutations most commonly arise in the primary target (parC of topoisomerase IV), producing low-to-moderate increases in minimum inhibitory concentration (MIC). Subsequent mutations in *gyrA* (DNA gyrase) then confer high-level resistance [48,52]. Mutations in *gyrA* and *parC* are far more common in clinical isolates than those in *gyrB* or *parE*. These amino acid substitutions reduce fluoroquinolone binding affinity to the enzyme-DNA complex, allowing DNA replication to continue in the presence of the drug.

An additional mechanism involves active efflux, which lowers intracellular drug concentrations. The main efflux systems in *S. pneumoniae* are the ABC transporters PatAB and PmrA [49]. Overexpression of these pumps, often due to mutations in their regulatory regions, contributes to elevated MICs. Plasmid-mediated resistance via Qnr proteins, which protect DNA gyrase and topoisomerase IV, has been described but appears to have limited clinical relevance in *S. pneumoniae* [49].

### 2.4. Other Antimicrobial Resistance Classes

The introduction of pneumococcal conjugate vaccines (PCVs) has substantially reduced the burden of resistant strains by decreasing the circulation of vaccine-type serotypes commonly associated with antimicrobial resistance [29]. However, serotype replacement has enabled the global expansion of certain non-vaccine serotypes, many of which exhibit high levels of resistance [24].

The evolution and spread of resistance in *S. pneumoniae* are closely linked to integrative and conjugative elements (ICEs), particularly those of the Tn916 family. These mobile genetic elements serve as key vehicles for the horizontal dissemination of multiple resistance determinants. For example, tetracycline resistance is primarily mediated by the *tetM* gene, which encodes the ribosomal protection protein TetM [34]. Resistance to trimethoprim–sulfamethoxazole results from alterations in the target enzymes dihydropteroate synthase (encoded by *folP*, also known as *sulA*) and dihydrofolate reductase (encoded by *folA*). These alterations typically involve point mutations and, in the case of FolP, amino acid insertions that are frequently observed in resistant clinical isolates [24,34].

Antimicrobial resistance in *S. pneumoniae* remains a dynamic global challenge. The application of advanced genomic tools is essential for ongoing epidemiological surveillance and for deciphering the molecular mechanisms that drive resistance. These insights are critical for developing targeted therapeutic strategies and optimizing next-generation vaccines. In parallel, antimicrobial stewardship programs and strengthened immunization efforts are vital to preserving the effectiveness of existing antimicrobials against pneumococcal infections [24].

## 3. Virulence Factors in Resistant Clones

The pathogenicity of *S. pneumoniae* is shaped by a complex interplay between antimicrobial resistance (AMR) and its virulence repertoire. Resistance to β-lactams is primarily driven by recombination events and point mutations in the penicillin-binding protein genes (*pbp1a*, *pbp2b*, and *pbp2x*), which produce low-affinity PBPs and promote the clonal expansion of successful lineages such as CC271, CC876, and CC81. Historically, an inverse relationship or evolutionary trade-off has been proposed, whereby highly invasive serotypes tend to remain antibiotic-susceptible, while resistant lineages are better adapted for persistence under selective pressure [53,54]. However, high-impact clones such as Spain23F-1, Taiwan19F-14, and Spain6B-2 challenge this paradigm by combining altered PBPs with macrolide resistance mechanisms (e.g., *erm*-mediated ribosomal methylation and *mef*-mediated efflux) and mutations in DNA topoisomerases (GyrA and ParC) that confer fluoroquinolone resistance [55].

The CC271/320 clonal complex, predominantly linked to serotypes 19F and 19A, exemplifies the successful convergence of antimicrobial resistance and virulence. These lineages display multidrug resistance to macrolides, β-lactams, and tetracyclines [56,57]. Despite widespread use of pneumococcal conjugate vaccines (PCV7 and PCV13), CC271 has persisted and expanded, partly through capsular recombination. Virulence determinants such as pneumolysin (*ply*), choline-binding protein A (*cbpA*), and type 1 and 2 pili are more frequently detected in these resistant clones compared with susceptible lineages, contributing to their predominance in invasive pneumococcal disease (IPD) [58]. This persistence is further supported by the integration of resistance determinants into mobile genetic elements of the Tn916 and Tn5253 families, which often co-carry *erm*(B) and *tet*M. These elements are associated with enhanced nasopharyngeal colonization and biofilm formation [49,58,59].

In Latin America, macrolide resistance is predominantly mediated by *erm*(B), frequently carried on Tn916-like integrative and conjugative elements. Invasive serotypes 19A and 6A show a strong association between high-level antimicrobial resistance and virulence determinants that enhance clinical fitness [37]. In Lima, Peru, macrolide resistance reached 61.3% after PCV13 introduction, driven by the increasing prevalence of *erm*(B) and *mef*(A/E) genes in emerging serotypes 19A and 6C. In these lineages, the polysaccharide capsule remains a critical factor for immune evasion [60]. In Paraguay, analysis of 793 IPD cases revealed high rates of severe disease (pneumonia 74.9%, meningitis 18.4%), with penicillin resistance reaching 32.2% among meningitis isolates. Serotype 14, linked to the international clone England14-9, has emerged with resistance to erythromycin (29.8%) and tetracycline (42.5%), associated with altered PBPs (PBP1a, PBP2x, PBP2b) and the presence of *erm*(B) and *mef*(E).

Similar patterns are observed in Indonesia, where 52.2% of isolates carry *erm*(B) and 30% harbor *mef*(A), with 17.8% showing co-occurrence of both genes. These determinants are strongly associated with serotypes 19F, 19A, and 6A/6B, which exhibit robust clonal expansion in both carriage and IPD. In China, genomic studies indicate that *erm*(B)-mediated macrolide resistance is commonly linked to multidrug resistance via Tn916-family elements, with serotypes 19A and 19F predominating. Their capsular polysaccharides facilitate immune evasion and support the dissemination of highly fit lineages, particularly in healthcare settings [43]. Comparable findings have been reported in Iran, where invasive isolates harboring *erm*(B) and *mef*(A/E)—mainly serotypes 19A and 23F—display a robust virulence profile characterized by consistent detection of key determinants including *cbpA*, *cpsA*, and *lytA* [61].

In Europe, Madrid has documented a resurgence of penicillin resistance (33.3%) among invasive isolates, driven by clones such as ST6521 (serotype 11A) and 19A lineages that combine macrolide and β-lactam resistance [62]. In Croatia, macrolide resistance in adults reached 32.1%, primarily mediated by *erm*(B) (MLS phenotype) in highly virulent clones such as ST320 (19A) and ST156 (9V) [63].

Antimicrobial resistance is not an isolated phenomenon, extending even to vulnerable populations, such as adults living with HIV. Among penicillin-non-susceptible *S. pneumoniae* (PNSP) isolates, 50% showed erythromycin resistance, with a balanced distribution between MLS (54%) and M (46%) phenotypes; all isolates carried the corresponding resistance genes [64]. The ability of *S. pneumoniae* to maintain this extensive repertoire of resistance determinants without compromising invasive potential highlights the importance of genomic surveillance that extends beyond conventional phenotypic characterization.

Collectively, these findings demonstrate that the global success of pneumococcal clones is driven by coordinated genetic mechanisms that tightly couple antimicrobial resistance with enhanced fitness, transmissibility, and virulence.

## 4. Clinical Impact of Antibiotic-Resistant and Virulent *Streptococcus pneumoniae*

Resistant clones frequently co-harbor virulence factors that increase morbidity, mortality, and the risk of treatment failure, particularly in pediatric and adult populations. Recent studies indicate that post-vaccine selective pressure, clonal expansion, and metabolic adaptations further exacerbate these threats [65].

Serotype distribution and clonal lineages strongly influence the interplay between antimicrobial resistance and virulence, thereby affecting clinical severity. In adult IPD cases from a Croatian tertiary hospital (2022–2025), serotype 3 predominated (34.1%, 29/85 isolates), followed by 19A (25.9%, 22/85). Isolates with reduced susceptibility to penicillin were enriched among serotypes 19A and 19F, with three of eight 19A isolates (37.5%) assigned to the globally disseminated ST320 clone. This lineage is notable for combining high virulence with a multidrug-resistant (MDR) phenotype [63]. Overall, MDR prevalence reached 15.3%, rising to nearly 50% among penicillin-non-susceptible isolates, which predominantly exhibited the erythromycin–clindamycin–tetracycline resistotype. These findings underscore the role of the 19A–ST320 lineage in driving treatment-refractory IPD in adults.

Similar patterns are observed in pediatric cohorts. In Northeast China (2000–2021), analysis of 1454 *S. pneumoniae* isolates (568 invasive, 886 non-invasive) identified serotypes 19F, 23F, 14, and 6B as predominant. Clonal complex 271 (CC271) was the dominant multilocus sequence type (MLST), and serotypes 19A and 19F accounted for the majority of penicillin-resistant strains. Notably, non-invasive isolates exhibited higher β-lactam resistance than invasive isolates. All strains remained susceptible to ertapenem, moxifloxacin, linezolid, and vancomycin. Resistance to macrolides and tetracycline was nearly universal, and the erythromycin–clindamycin–tetracycline–trimethoprim/sulfamethoxazole resistotype was common among MDR isolates [66]. Expression of virulence-associated genes (*cbpA, rrgA, psrP*) was lowest in serotype 14 but comparable between serotypes 19A and 19F, supporting their association with both invasive potential and antimicrobial resistance.

Post-PCV dynamics further highlight shifting clinical burdens. In Poland, the introduction of PCV10 into the National Immunization Program in 2017 reduced the proportion of PCV10 vaccine-type (VT) strains in children < 2 years from 56.9% (pre-PCV, 2014–2016) to 29.5% (post-PCV, 2017–2020) [67]. Concurrently, non-VT serotypes increased from 12.8% to 22.9%, while additional PCV13 serotypes (3, 6A, 19A) rose from 17.6% to 25.7%. Resistance rates declined overall, with penicillin resistance decreasing from 49.0% to 34.3%, erythromycin resistance from 55.9% to 41.9%, and MDR from 46.1% to 30.5%. The proportion of piliated isolates—associated with enhanced epithelial adherence and increased virulence—declined from 50.0% to 28.6% [67]. In parallel, the distribution of Global Pneumococcal Sequence Clusters (GPSC) shifted, with GPSC1 predominating pre-PCV (17.6%) and GPSC12 increasing post-PCV (10.5%) [67]. These reductions in resistant and piliated strains were associated with decreased IPD caused by highly virulent, MDR clones, highlighting the beneficial role of vaccination in mitigating clinical burden.

In urban Malawi, 4–8 years after PCV13 rollout, whole-genome sequencing of 2804 nasopharyngeal carriage isolates identified 148 discrete metabolic genotypes (MTs). Emerging MTs (e.g., MT109/serotype 17F, MT93/23B, MT120/38) exhibited distinct virulence and antimicrobial resistance (AMR) profiles. Functional characterization using in vitro and murine models confirmed phenotypic differences: MT93 (23B) showed increased epithelial invasion (*p* = 0.03) and haemolysis (*p* = 0.03) compared with ancestral MTs, whereas MT120 demonstrated enhanced murine colonization (competition index *p* < 0.001). Pan-genome-wide association analyses identified genes related to metabolism, cellular processes, and AMR/virulence (all Bonferroni-adjusted *p* < 0.0001) as drivers of lineage expansion [13]. Collectively, these findings indicate metabolic–virulence–AMR co-evolution, suggesting ongoing post-vaccine adaptation that may sustain carriage and contribute to IPD resurgence.

Regional genomic surveillance further documents the emergence of hypervirulent resistant clades. In Shenzhen, China (2009–2017), IPD isolates from children were dominated by clonal complex 320 (CC320), primarily associated with serotypes 19A and 19F. A novel subclade, CC320_SZpop (ST271), exhibited increased β-lactam tolerance and enhanced pathogenicity compared with imported ST320 strains. Unique virulence determinants were identified in CC320_SZpop, along with an approximately 40 kb recombination hotspot that may facilitate the acquisition or loss of AMR genes [68]. During the 2022 COVID-19 wave in Southwest China, the proportion of pediatric IPD isolates belonging to serotype 14 was significantly higher in invasive disease compared with non-invasive pneumococcal disease (NIPD) (*p* < 0.05). Invasive strains exhibited high levels of penicillin non-susceptibility under meningitis breakpoints (100% resistant) but lower resistance rates under non-meningitis criteria. Serotype 19F displayed distinct AMR and virulence gene profiles [69].

Novel sequence types further signal evolving clinical threats. In Kuwait (2018), analysis of 31 IPD isolates identified 28 STs, including 14 novel STs (45.2%). Serotype 8-ST53, a highly virulent lineage, and serotype 19A associated with novel STs were predominant. Many isolates exhibited resistance to erythromycin and azithromycin, with MDR particularly common among blood isolates. High singleton diversity (83.9%) and genetic relatedness to strains circulating in neighboring Gulf countries suggest ongoing local recombination and capsular switching, processes that may compromise vaccine effectiveness [70].

A global pan-genome-wide association study of 1292 serotype 19A isolates from patients with invasive disease and asymptomatic carriage identified 30 consensus disease-associated genes using three orthogonal approaches (Scoary, linear mixed model, and random forest). These genes encode functions related to mobile genetic elements, AMR, virulence, and metabolism, highlighting the multifactorial basis of pathogenicity in this hypervirulent serotype despite PCV13-associated selective pressure [71].

Collectively, these data demonstrate that resistant and virulent pneumococci continue to impose a substantial clinical burden through increased IPD severity, treatment challenges, and post-vaccine lineage replacement. High-risk clones such as ST320 and CC271, along with emerging metabolic types (MTs), link MDR phenotypes with enhanced adherence, invasion, and colonization, thereby increasing the risks of meningitis, bacteremic pneumonia, and prolonged hospitalization. Although PCVs have reduced vaccine-type disease and associated resistance and virulence traits in some settings, the expansion of non-vaccine-type lineages and ongoing metabolic adaptation threaten to erode these gains. Accordingly, continuous genomic surveillance, the development of updated vaccines targeting conserved virulence determinants, and strengthened antimicrobial stewardship are essential to mitigate future clinical impact.

## 5. Virulence–Resistance Coevolution: From Classical Trade-Offs to Contemporary Success

The Classical Paradigm and Its Evolutionary Basis. The coexistence of virulence and antimicrobial resistance (AMR) within a single bacterial lineage represents a fundamental paradox in microbial evolution. The classical fitness cost hypothesis posits that resistance determinants impose a significant metabolic burden on bacteria, typically manifesting as reduced growth rates, impaired transmissibility, or attenuated pathogenicity in the absence of antibiotic selective pressure. It is widely accepted that such fitness costs play a central role in shaping the maintenance and evolutionary stability of both virulence and AMR traits. From an evolutionary perspective, selection favoring enhanced virulence is generally expected to occur at the expense of antimicrobial resistance (or vice versa), unless compensatory mutations or genetic mechanisms arise to alleviate these costs and restore overall fitness [72,73,74].

In *S. pneumoniae*, empirical support for the virulence–resistance trade-off was first established in murine infection models, which demonstrated a positive correlation between penicillin minimum inhibitory concentration (MIC) and LD50 in serotype 6 isolates [75]. This was later mechanistically linked to allelic replacement of pbp2x and pbp2b, showing that selection for resistance directly reduced virulence [76]. At the epidemiological level, an inverse relationship is evident in Spain, where ST62 isolates (serotype 11A) were penicillin-susceptible yet displayed enhanced resistance to opsonophagocytosis compared with less virulent counterparts [77]. Collectively, these observations support the notion that more virulent strains tend to exhibit greater antimicrobial susceptibility.

Epistasis Modulates Fitness Costs. A key factor influencing the fitness impact of AMR is epistasis, whereby the phenotypic effect of a mutation depends on the genetic background of the organism [78]. Epistatic interactions can either exacerbate or alleviate fitness costs. In *S. pneumoniae*, compensatory epistasis has recently been documented: while individual mutations in *parC* or *gyrA* reduced virulence (increased mouse survival), the simultaneous presence of both mutations restored fitness to wild-type levels. This indicates that epistatic interactions between resistance determinants can generate compensatory mechanisms that stabilize virulence without compromising resistance [79]. Consequently, the fitness cost of resistance in pneumococcus is not fixed but dynamic, shaped by the broader genetic landscape. This phenomenon helps explain why certain multidrug-resistant lineages maintain high competitive fitness in clinical settings, as illustrated in the following examples.

Contemporary Lineages That Overcome the Trade-Off. Modern pneumococcal lineages have emerged that successfully combine high virulence and antimicrobial resistance, effectively bypassing the classical trade-off. In Malawi, the Global Pneumococcal Sequence Cluster 10 (GPSC10) ST700 lineage (serotype 3) harbors gene clusters for nutrient uptake and bacteriocin production. It carries six deletions in the capsular polysaccharide (*cps*) locus that, surprisingly, do not impair capsule production but instead enhance resistance to opsonophagocytosis relative to other serotype 3 strains. This lineage also carries *tetM* and exhibits predicted penicillin non-susceptibility (MIC > 0.5 μg/mL), thereby coupling enhanced AMR with superior immune evasion [80]. Similar success is seen in GPSC10 (serotype 24F), which combines high invasive potential, extensive capsular switching across at least 17 serotypes, and multidrug resistance [81]. Additional examples include ST271 (predominantly serotype 19F) and CC320 (19F/ST271), which display reduced β-lactam susceptibility alongside elevated prevalence of virulence-associated genes [68,82]. These patterns align with the epidemiological dominance of serogroup 19 as the leading invasive serogroup in post-PCV surveillance, frequently linked to MDR [83,84], enabling coexistence of high virulence and AMR within the same lineage.

The Invasiveness–Resistance Relationship at the Epidemiological Level. Clinical outcomes in IPD are modulated by host factors such as age and comorbidities, yet at the bacterial level they are driven by strain-specific genetic and phenotypic traits [12,85]. Using serotype alone as a predictor of invasiveness is therefore limited, as IPD arises from complex host–pathogen interactions. Regarding antibiotic resistance, IPD isolates are generally less resistant than carriage or non-invasive isolates (Table 1). However, this relationship is not uniform across the pneumococcal population and varies markedly by serotype. A retrospective analysis of isolates from China found that serotypes 19F and 19A were the most penicillin-resistant but least invasive, whereas serotypes 4 and 8 were highly invasive yet least resistant [54]. These data reinforce a negative correlation between penicillin resistance and invasiveness in *S. pneumoniae*. Comparable patterns have been observed in other pathogens, such as *Salmonella enterica*, where AmpC β-lactamase expression is associated with reduced invasive capacity [86]. Although the precise molecular mechanisms remain incompletely understood, the metabolic burden of resistance determinants may impair the bacterium’s ability to invade diverse host niches. This suggests a new paradigm in which antimicrobial resistance and invasive potential are biologically interconnected rather than independent traits.

Non-Encapsulated *S. pneumoniae* and Antimicrobial Resistance. The pneumococcal capsule is a major virulence factor that shields the bacterium from opsonophagocytosis and facilitates nasopharyngeal colonization [19]. Acapsular mutants accordingly show reduced virulence in murine models [93]. Nevertheless, non-encapsulated *S. pneumoniae* (NESp) can cause conjunctivitis and otitis media, indicating alternative virulence strategies. One such mechanism involves expression of the pneumococcal surface protein PspK, which promotes epithelial cell adherence [94]. Notably, multidrug resistance is a common feature of NESp strains [19,95]. Consistent with this, South African data show higher penicillin resistance rates in non-invasive pneumococcal disease (NIPD) than in IPD isolates (Table 1). From a genome plasticity perspective, capsule loss appears to be compensated by acquisition of alternative fitness traits, including enhanced antimicrobial resistance. Recent evidence further suggests that the negative correlation between resistance and invasiveness observed in encapsulated strains also extends to NESp populations.

## 6. Future Therapeutic and Preventive Strategies

Current therapeutic approaches for multidrug-resistant *S. pneumoniae* (MDR-Spn) rely largely on broad-spectrum agents, including glycopeptides and fluoroquinolones. In recent years, several novel antimicrobials have been developed for the treatment of community-acquired bacterial pneumonia in adults, some of which demonstrate activity against MDR-Spn.

Omadacycline, an aminomethylcycline, is a semisynthetic tetracycline derivative characterized by a modification at the C-9 position of the D-ring with an aminomethyl group. This structural alteration enables it to overcome common tetracycline resistance mechanisms, including ribosomal protection mediated by *tet*(*M*), *tet*(*O*), and *tet*(*S*), as well as efflux mechanisms mediated by *tet(K*), *tet*(*L*), *tet*(*A*), and *tet*(*B*) [96,97]. In vitro, omadacycline demonstrates high activity against *S. pneumoniae* (MIC_50/90_, 0.06/0.12 mg/L; 98.7% susceptible), representing at least a 64-fold lower MIC compared with tetracycline (MIC_50/90_, >4/>4 mg/L) [98]. It was approved by the U.S. Food and Drug Administration (FDA) in 2018 for the treatment of adults with community-acquired bacterial pneumonia, based on clinical data demonstrating noninferiority to moxifloxacin [99,100].

Eravacycline further expands therapeutic options for MDR-Spn, particularly in cases where resistance to earlier tetracyclines limits treatment choices, especially in patients with intra-abdominal infections [101,102]. Lefamulin, a novel pleuromutilin antimicrobial, inhibits bacterial protein synthesis by selectively binding to the peptidyl transferase center of the 50S ribosomal subunit and exhibits limited cross-resistance with other ribosome-targeting agents [103]. It demonstrates potent in vitro activity against MDR-Spn, with MIC_50/90_ values around 0.12/0.25 µg/mL in global surveillance studies [104,105]. Lefamulin was developed for the treatment of community-acquired bacterial pneumonia and has shown early clinical response rates comparable to moxifloxacin (87.3% vs. 90.2%), confirming its noninferiority. It was approved by the FDA in 2019 [106].

Nemonoxacin, a non-fluorinated quinolone, demonstrates potent in vitro activity against MDR-Spn, including strains harboring fluoroquinolone resistance [88,107]. Contezolid, a next-generation oxazolidinone, has been developed to improve the safety profile of linezolid. It exhibits strong in vitro activity against Gram-positive pathogens, including Spn. A multicenter study evaluating in vitro antimicrobial activity reported a low resistance rate of 1.9% (4/208 isolates) [88].

Several therapeutic strategies beyond conventional antimicrobials are being explored for future clinical application against MDR-Spn. Phage therapy represents a promising approach for the treatment of MDR bacterial infections, including Spn. Although multiple bacteriophages capable of infecting Spn have been identified [108], research has increasingly focused on phage-derived endolysins. Cpl-1, one of the most extensively studied pneumococcal endolysins produced by the bacteriophage Cp-1, exhibits potent lytic activity against a broad range of serotypes [109]. It demonstrates synergistic antimicrobial effects when combined with antimicrobials [110] and can restore susceptibility to β-lactam and macrolides in resistant pneumococci in vitro [111]. Furthermore, Cpl-1 has shown efficacy in animal models, providing protection against nasopharyngeal colonization [112], pneumonia [113], and invasive pneumococcal diseases [110,114,115].

Recent studies have further expanded the therapeutic potential of bacteriophage-derived endolysins. Emerging delivery strategies, including mRNA-encoded endolysins, enable host cells to produce bactericidal lysins targeting *S. pneumoniae* [116]. In addition, a novel CHAP-domain-containing pneumococcal endolysin, SP-CHAP, has demonstrated enhanced activity compared to Cpl-1 against both planktonic and biofilm-associated pneumococci and has reduced nasopharyngeal colonization in vivo [117]. Despite these promising preclinical findings, clinical trials evaluating bacteriophage-derived endolysins against Spn have not yet been reported. Further translational and clinical investigations are therefore warranted.

Monoclonal antibodies (mAbs) represent an emerging strategy for the prevention and treatment of MDR-Spn infections. Current research has focused on antibodies targeting key pneumococcal virulence factors, including toxins, surface proteins, and capsular polysaccharides. Monoclonal antibodies directed against toxins can neutralize toxin-mediated host damage. Among these, anti-pneumolysin mAbs are the most extensively studied in preclinical models. Previous studies have shown that these antibodies inhibit pneumolysin-mediated hemolysis in vitro and reduce pulmonary colonization in vivo [118,119].

A recent study reported a humanized mAb targeting pneumolysin, 2E5zumab, which demonstrated a prolonged half-life and high bioavailability in a murine model [120]. Conserved pneumococcal surface proteins, including PhtD, PspA, and PcpA, represent promising targets for mAb-based therapies. Antibodies directed against these proteins enhance opsonophagocytic killing and promote complement-mediated bacterial clearance [121]. In murine models, human anti-PhtD and anti-PspA antibodies have shown robust opsonophagocytic activity and prolonged survival [122]. Similarly, antibodies targeting capsular polysaccharides mediate opsonophagocytosis and complement activation [123]. However, because these responses are often serotype-specific, many studies have focused on serotypes less effectively covered by current vaccines (e.g., serotype 3), although mAb combinations may broaden coverage [123,124]. Consequently, recent research has increasingly shifted toward targeting conserved pneumococcal antigens rather than serotype-specific capsular components. To date, clinical trials evaluating these therapeutic antibodies in humans have not been reported, underscoring the need for further translational and clinical investigation.

Antimicrobial peptides, such as the human cathelicidin LL-37, have demonstrated antimicrobial activity against *S. pneumoniae* by destabilizing the bacterial membrane [125]; however, LL-37 has also been reported to induce MefE/Mel-mediated macrolide resistance [126]. More recently, alternative therapeutic strategies targeting the bacterial membrane have emerged, including antimicrobial photosensitizers and antimicrobial photodynamic therapy [127].

Vaccination strategies remain central to the prevention of Spn infection. The introduction of pneumococcal conjugate vaccines (PCVs) has reduced the prevalence of antimicrobial-resistant pneumococci [128]; however, serotype replacement continues to drive the emergence of resistant non-vaccine serotypes [129]. In 2024, the FDA approved PCV21 for use in adults. Unlike earlier conjugate vaccines such as PCV20, PCV21 was designed to target additional non-vaccine serotypes that have expanded due to serotype replacement, including antimicrobial-resistant serotypes such as 15A and 23A [130]. Next-generation vaccine approaches—including protein-based vaccines [123], whole-cell vaccines [131,132], and higher-valent conjugate formulations [133] (37)—are currently under development to address the spread of MDR-non-vaccine serotypes.

In addition to these innovative strategies, several other emerging approaches are being investigated for MDR-Spn, including host-directed therapies [134], anti-toxin strategies [135], and gene-targeting approaches [136]. Although these modalities hold promise for future clinical application, continued antimicrobial stewardship remains essential [137]. Ultimately, a multifaceted approach integrating optimized antimicrobial use with emerging therapeutic and preventive interventions will be required to mitigate the global threat posed by MDR-Spn.

## 7. Conclusions and Research Priorities

The convergence of antimicrobial resistance (AMR) and virulence in *S. pneumoniae* represents a highly effective evolutionary strategy. Through mosaic PBP genes, mobile genetic elements of the Tn916 family, and capsular recombination, clones such as CC271/320 have successfully combined resistance to β-lactams, macrolides, and tetracyclines with enhanced virulence traits, including pneumolysin, CbpA, pili, and robust capsule expression. These adaptations have enabled persistence and expansion in the post-PCV era across multiple continents.

This resistance–virulence linkage drives substantial IPD burden, treatment failures, and mortality, particularly in children, older adults, and immunocompromised populations. The 2024 WHO BPPL update classifying macrolide-resistant *S. pneumoniae* as a medium priority pathogen underscores its public health importance amid serotype replacement and non-vaccine serotype expansion.

Although PCVs and antimicrobial stewardship have reduced resistance in some settings, emerging metabolic genotypes and non-vaccine lineages continue to adapt. Novel agents (omadacycline, lefamulin, eravacycline), phage-derived endolysins, monoclonal antibodies, and next-generation vaccines (e.g., PCV21 and protein-based formulations) offer promising tools. However, sustained progress requires strengthened surveillance, mechanistic research on AMR–virulence synergies, and equitable implementation of interventions.

Key research priorities include: (1) expanded global genomic and metabolic surveillance, especially in low- and middle-income countries; (2) clinical trials of novel therapeutics and broader vaccines; (3) deeper investigation of metabolic adaptations and epistatic interactions; and (4) integrated One Health antimicrobial stewardship. A multifaceted approach combining surveillance, innovation, and responsible antibiotic use is essential to curb the threat of resistant pneumococcal disease.

## Figures and Tables

**Table 1:** Comparison of antimicrobial resistance profiles between IPD and non-invasive IPD (NIPD) *S*. *pneumoniae* isolates across different countries.

Country (Period)	Sero-Type	n	IPD		NIPD		Breakpoint Criteria (CLSI)	Pattern	Reference
			%	Resistance Profile (%)	%	Resistance Profile (%)			
China (NR)	19F	189	21.5	PEN-NS (100)	78.4	PEN-NS (97.4)	NR	IPD ≈ NIPD	[87]
China (January 2023–June 2024)	—	208	25.9	PEN-R (0)	74.0	PEN-R (4.0)	NR	NIPD > IPD	[88]
China (January 2000–December 2021)	—	1454	39.0	PEN-R (17.5)	60.9	PEN-R (24.2)	non-meningitis	NIPD > IPD	[66]
China (NR)	19F	172	2.9	PEN-NS ^✤^ (62.8)	97.1	NR	NR	~	[54]
China (NR)	4	5	80.0	PEN-NS ^✤^ (0)	20.0	NR	NR	~	[54]
Australia (August 2018–December 2021)	—	1470	87.4	PEN-R (20.3)	12.5	PEN-R (31.4)	meningitis	NIPD > IPD	[89]
United States (January 2011–February 2020) ^1^	—	3012 ^a^ 3037 ^b^	12.6	PEN-R (20.6) MAC-R (31.7)	87.3	PEN-R (42.4) MAC-R (41.1)	NR	NIPD > IPD	[29]
United States (January 2011–February 2020) ^2^	—	7516 ^a^ 12,847 ^b^	39.0	PEN-R (14.4) MAC-R (27.2)	60.9	PEN-R (27.0) MAC-R (44.5)	NR	NIPD > IPD	[90]
South Africa (2003–2013)	NESp	172	22.6	PEN-NS (35.9) ERY-NS (30.8)	77.3	PEN-NS (76.7) ERY-NS (52.6)	meningitis ^✯^	NIPD > IPD	[91]
Israel (January 1995–May 1999)	6B	64	17.1	PEN-R (27.7) ERY-R (27.7)	82.8	PEN-R (57.0) ERY-R (60.0)	NR	NIPD > IPD	[92]
Israel (January 1995–May 1999)	23F	67	22.3	PEN-R (80.0) ERY-R (0)	77.6	PEN-R (92.0) ERY-R (12.0)	NR	NIPD > IPD	[92]

Abbreviations and notes: IPD, invasive pneumococcal disease; NIPD, non-invasive pneumococcal disease; NESp, non-encapsulated *Streptococcus pneumoniae*; PEN, penicillin; ERY, erythromycin; MAC, macrolide class; PEN-R, penicillin-resistant; PEN-NS, penicillin-non-susceptible; ERY-R, erythromycin-resistant; ERY-NS, erythromycin non-susceptible; n, number of tested isolates; ^a^, number of tested isolates for PEN; ^b^, number of tested isolates for MAC; ^1^, study was performed in children (<18); ^2^, study was performed in adults (≥18); NR, not reported by original study; —, data not available or not applicable. All resistance categories follow CLSI breakpoints as reported in the original publications. ^✤^, Original publication used the term “insensitivity”, standardized here to “non-susceptibility” per CLSI nomenclature; ^✯^, breakpoint was only applied in penicillin. The pattern column summarizes the relative resistance profile: **NIPD > IPD** indicates higher resistance in non-invasive isolates; **IPD ≈ NIPD** indicates comparable resistance between groups; ~ indicates no comparison is possible due to missing NIPD data. Of the 11 comparisons, 8 show NIPD > IPD, 1 shows IPD ≈ NIPD, and 2 lack NIPD data, supporting the trend that non-invasive isolates frequently exhibit higher resistance than invasive ones.

## Data Availability

No new data were created or analyzed in this study. Data sharing is not applicable to this article.
